# Relieving one’s relatives from the burdens of care

**DOI:** 10.1007/s11019-017-9815-9

**Published:** 2017-11-30

**Authors:** Govert den Hartogh

**Affiliations:** Department of Philosophy, University of Amsterdam, Staten Bolwerk 16, 2011 Haarlem, ML The Netherlands

**Keywords:** Burdens of care, Reception of care, Duty to die, Altruistic suicide, Obligations of family members

## Abstract

It has been proposed that an old and ill person may have a ‘duty to die’, i.e. to refuse life-saving treatment or to end her own life, when she is dependent on the care of intimates and the burdens of care are becoming too heavy for them. In this paper I argue for three contentions: (1) You cannot have a strict duty to die, correlating to a claim-right of your relatives, because if they reach the point at which the burdens of care are larger than you can reasonably expect them to take, the natural conclusion is that their duty ends. (2) They may be prepared, however, to go on caring for you beyond that point. In that case your responsibility for their wellbeing may require you to refuse this care, even if this results in a situation for you in which death will be preferable to continued life. (3) If this is the correct understanding of your responsibilities, the objection that in the context of family life the burdens of care attached to one family member’s valued existence can never be ‘too heavy’, fails. It postulates unlimited concern on one side and a total lack of concern on the other.

Most people, in the richer countries anyway, die these days after a prolonged period of illness, either a lethal illness that can at best only temporarily be halted, or a cumulation of debilitating old age ailments. Their physical and mental capacities decline and they are in need of care to go on functioning, in many cases at some point intensive care, up to 24 h a day. This care is often provided by relatives, mostly a partner or children, sometimes by friends or other volunteers. But it is also often given by professional care providers, either in a hospital setting or not. Often the costs of this professional care have to be paid for, at least partially, by the care-receiver, but if his means are insufficient, relatives often have to stand in. The burden for the relatives involved in giving care themselves or in paying for it can be large, even in the European welfare states.

In the Netherlands, for example, about 1 million people (on a population of 17 million) provide long-term intensive care to someone else, usually a relative, on a voluntary basis, and according to one study almost half of them feel overburdened.^1^ In some cases this leads to neglect of duty, abuse and other derailments, but it may also have negative consequences for the caregiver’s own health, financial situation, family and professional life. Municipalities have a legal obligation to monitor informal care-giving and to provide support if this is needed, but the support they actually provide is often too late and too little.

In other countries the situation may be worse. In particular the financial burdens involved in the provision of institutional and professional care may be much heavier. [In the Netherlands these are dependent on the patient’s income and capped at € 2300 a month (2016).] In an extreme case it could occur that when someone finally dies, the person who has cared for her has in the meantime lost his money, his career, his partner, his house and his health.^2^ Should one allow such things to happen, if one is the person being cared for? Or does one have the duty to prevent it, by refusing treatment or if necessary by ending one’s own life?

My argument is meant to apply to both the physical care provided by relatives, and the financial care of paying the costs of the provision of physical care by others. Both kinds of care can become excessively burdensome.

My discussion will be limited to cases in which the recipient of the care is fully aware of its burdens. In most actual cases he will be cognitively impaired to such an extent that we cannot expect him to recognize any supposed ‘duty to die’ at the moment at which the burdens of care get too heavy. To decide whether, in that case, he could have a duty to prevent this situation from emerging would be the next step.

I will start (Why there normally cannot be any duty to die section) by arguing that you cannot have a strict duty to die, correlating to a claim-right of your relatives. However, your responsibility for their well-being may require you to refuse their care, even if this results for you in a situation in which death will be preferable to continued life. In the next section (What kind of requirement is this?) I discuss several possible interpretations of this requirement and conclude that it refers to a consideration that you don’t give its proper weight if you don’t refuse your relatives’ care. A complicating factor that I take into account next is that your refusal by itself may also have very adverse emotional consequences for your relatives. In a final section (Objections) I consider whether the objections that have been made to the idea of a ‘duty to die’ hold in regard to this particular specification, or rather modification, of that idea.

## Why there normally cannot be any duty to die

The discussion about such a duty, as it has been proposed by John Hardwig in particular,^3^ has been somewhat confused because it has been unclear what is meant by a ‘duty’ in this context. Could it be a strict or perfect duty that you may owe to your relatives? If so, they would have a corresponding claim-right to the fulfilment of the duty, a right that they could also waive, if they wished. Hardwig has denied that he meant such a perfect duty, he prefers to speak of a ‘personal responsibility’. But how can this be, critics have asked.^4^ The reason why you are supposed to have this responsibility is that you are causing harm to your relatives, more than you can reasonably expect them to bear. Doesn’t this imply that, if it is wrong to expect them to take that burden, it is wrong *to them*? If so, your duty to relieve them of that burden can only be a duty you owe to them.

But such a duty cannot exist. Either your relatives owe you a duty of care, then they cannot require you to relieve them from the burdens involved. Or they have no such duty. Then it makes no sense for them to claim that you relieve them from the burdens of care by ending your life. When the burdens of care are getting too heavy, they can, instead, stop caring and leave the consequences to you. It would be perverse for them to require you to save them the emotional costs of stopping to care by stopping being there as a possible object of care. How could we make sense of those ‘emotional costs’, if they are not rooted in a concern for your welfare?

Suppose that in a certain country old people, when they can no longer take care of themselves and become a danger for their surroundings, are transferred to a nursing-home and, if they are unable to pay for this care from their own pockets, the costs of their stay are automatically charged to their relatives, without any upper limit. It would mean that the state might *force* these relatives to lose their money, career, partner, home and health. In such an institutional context it would make sense to talk about a strict duty to end one’s life. It may be true, as critics allege, that this would have very negative consequences for family life, but these consequences should then be attributed to the institutional arrangement. The present trend in European welfare states to relocate duties of care from the state to the family may already start to have some such consequences.

Outside such weird institutional environments there cannot be any proper duty to die, because it is inconsistent to attribute to anyone at the same time an unlimited duty of care, and a claim-right to be freed from its burden by the elimination of its recipient. At the very least, in stead of choosing to kill oneself, one could always waive one’s supposed right to the care of others. But it is more proper to say that, if there is such a right, it has some inherent limits. It may be true that, if you have no claim to the care of others, and they in fact do not intend to do more than their duty, the inevitable consequence for you will be death, but that still doesn’t mean that you have a duty to die.^5^

How do we determine the inherent limits of the relatives’ duty of care? One could suggest that the duty ends when its burdens to the care-giver outweigh its benefits to the recipient. In general this requirement is much too strong, but family members are usually supposed to have more extensive obligations to each other than others. On most accounts, however, the limits of your relatives’ duty of care are not only determined by your needs and their own capacities. A plausible view is that the extent of any duty of family members to each other depends on the way they have mutually defined their relation through the history of their interaction. Only parents have duties of care to the people they have put into existence that do not fully depend on the actual development of their relation.^6^ It is true that, even if the obligations of relatives to each other are historically contingent in this way, at any particular moment they are a moral fact that is not open to instant renegotiation. But, whatever their history, they are never unlimited.

Of course your relatives are free to go on caring for you beyond those limits. And precisely because these limits are not only dependent on their capacities but on many other factors as well, the fact that they have reached the upper limits of their duty doesn’t necessarily imply that they will be overburdened by continuing to care. That doesn’t mean, however, that you need not be concerned at all about the burdens involved in that care. Either at this or at some later time you may have to conclude that these burdens are growing excessive and that, out of concern for their welfare, you shouldn’t allow them to go on.^7^

Doesn’t this mean that you really *have* a ‘duty to die’ of sorts after all, even if it isn’t a perfect duty that you owe to your relatives? It is common and not improper to say of someone who has decisive moral reasons to act in a certain way that she has the ‘duty’ to act in that way, but I prefer to avoid that language.^8^ Such a ‘duty’ is not a perfect duty owed to someone who has a correlating claim-right. The substantial question, however, is whether, even if you cannot have a strict duty to end your life, you can have decisive moral reasons to do so in the interest of others.

In order to answer that question, we should distinguish between several possible scenario’s. Suppose first that if your relatives stop providing you the care they used to give, your condition though significantly worse than before, will still be such that you prefer to go on living. In that case you need only to agree to the cessation of the existing arrangement, or, if necessary, to refuse its continuation. You may for example insist on being transferred to a nursing home.

Suppose, secondly, that your condition will be such that you reasonably prefer death, and are prepared to act on that preference, either by refusing treatment or by suïcide. In that case we still have to distinguish between two decisions: your decision to refuse the care provided by your relatives and the decision to end your own life. Having made the first decision you can still reconsider the second one, going into that nursing home after all. Even in this second scenario case it cannot be said that the second decision is by itself other-regarding, you do not end your life *in order to* relieve your relatives from the burdens of care.

It is only when, thirdly, your very refusal of your relatives’ care means by itself that you inevitably die on the short term, and no second decision is needed, or only a decision concerning the exact timing and manner of your death, that your refusal can plausibly itself be seen as an altruistically motivated choice for death. Consider the well-known case of Captain Oates. It had been permissible for Oates’ comrades to leave him behind and let him die. They had no duty to endanger their own lives by taking him with them. Hence Oates did not have a strict duty to end his own life either, corresponding to a claim-right of his comrades that he did.^9^ He did have, however, decisive moral reasons not to allow them to endanger their own lives. To refuse to be taken along further was therefore the right thing for him to do, even though he would have violated no-one’s rights if he had allowed his comrades to go on taking him with them. Because the decision to stay where he was implied certain death, it was a decision to die.^10^ In this sense Oates acted on a moral requirmenet which can be properly described as a requirement to die.

Decisions in this third category are probably very rare, perhaps virtually non-existent in the Western world, at least in the context of care. In emergency cases you will not be left to your fate. If this is correct, the really interesting case is the second scenario, in which acting in a responsible way requires you to refuse your relatives’ efforts, knowing that this will lead to a situation in which refusing treatment or suicide will, for self-regarding reasons, be your best next choice.^11^

## What kind of requirement is this?

In all three scenario’s what you basically ought to do is to refuse the care your relatives are prepared to give you. In the first section we saw already that, excepting extreme circumstances, you have no proper duty to end your life in either of the scenario’s. For the same reasons you cannot have a proper duty to refuse your relatives’ care either. It makes no sense to ascribe to them a corresponding claim-right for your refusal, for, as we assumed, they are within their rights if they decide to stop caring, but are actually prepared to go on. Hence you are acting within *your* rights if you don’t refuse. In that sense you have the authority either to make that decision or not to make it. Bur you can exercise that authority in a responsible way or fail to do that. If you don’t make the decision to relieve them of the burdens of care because your absolute priority is to go on living, or (in the first secenario) living as comfortably as you presently do, you are lacking in proper concern for your relatives, primarily because you allow them to make unacceptable sacrifices for you but also because you shift on to them the agony of the decision to stop. Don’t they have a right to be shown proper concern? Maybe, but that is irrelevant. If they have such a right, they may be considered to have waived it in this case. But then you are still lacking in proper concern if you don’t refuse to be the recipient of their care. What is required by proper concern depends on their interests, not on their decisions.

How should we then understand the relevant notion of right and wrong decisions, if not in terms of proper duties? We could explain it in virtue-ethical terms: if you don’t refuse your intimates’ care when it threatens to destroy their lives, you are lacking in the relevant virtues of parents, partners, friends, etc. That is not incorrect, but the problem with it is that, by itself, it doesn’t capture the reasons of the virtuous person herself. The focus of her concern isn’t her own virtue, but the welfare of her children, partner, friend. Her motivating reason is not ‘I would be lacking in proper concern if I didn’t stop my daughter to be overburdened’, but ‘my daughter is overburdened’. The best explanation therefore is that if you don’t refuse your relatives’ care, there is a relevant other-regarding consideration that you don’t give its proper decisive weight. The point is that there are such considerations that aren’t considerations of (strict) duty.^12^ It is not improper, although less precise, to say that it would be wrong for you not to refuse your relatives’ care. It would be wrong, even if you would not be wronging *them*.

That it is only your personal responsibility to give this consideration its proper weight, not a duty you owe to anyone, doesn’t imply that others cannot criticize you for failing to do the right thing. That is not already ‘exercising a claim right’. But that you are criticizable for a moral failing at some point doesn’t mean that everyone is in an equally good position to reprimand you. In particular, if your relatives of their own free will make excessive sacrifices in caring for you, it would be improper for them, not only to claim it as a duty you owe to them to renounce those sacrifices, but also to criticize you for accepting them.

Isn’t it a form of objectionable paternalism if, out of a concern for their welfare, you interfere with the execution of decisions they have freely made? It isn’t because what you prevent by your refusal is those actions having a certain impact *on you*, and it is up to you to allow or to refuse your life to be determined by their decisions. It would therefore rather be paternalistic of them not to respect your refusal (in the end).

Hardwig’s own present view is that your responsibility (on his view the responsibility to end your life when the burdens of care for your relatives are getting too large) is implied by your personal commitment to take their interests into account.^13^ That, however, doesn’t seem quite right either, for it seems to assume that this commitment is optional. In that case you would, depending on your choice to accept or to reject this basic commitment, at best only have a conditional responsibility. We should rather say that you may have a categorical responsibility, but it should be left to you to recognize it and act on it. In a similar way you may have a debt of gratitude to someone, that on the one hand does not depend on your recognition of such a debt, but on the other hand cannot be claimed by your benefactor. For nothing you would pay in response to such a claim would express gratitude.^14^

Shouldn’t we rather say that it would be praiseworthy for you to refuse your relatives’ caring efforts, with all the consequences it has in the different scenario’s, but not blameworthy not to do so?^15^ Consider how your family would respond to such a ‘supererogatory’ act. Most likely they would reproach you for not permitting them to express their love to its full extent. Perhaps they would pity you for your neuroticism. They would have a point: if *they* are prepared to go a long way, it would be a kind of personal rejection not to allow them to go it.

That doesn’t mean that you have to go as far as they are prepared to go: at some point it would be wrong for you to accept further sacrifices. But below that point it might be equally wrong for you not to accept them. As Eva Feder Kittay puts it, “we have a moral responsibility to receive with grace care that is offered in good will and with the requisite competence.”^16^ Hence there is not much room for heroism in this area. Perhaps, if you are the kind of person for whom it is extremely hard to be dependent on the care of others, you should even be prepared to make some sacrifices in order to permit your relatives to make theirs.^17^

A highly complicating factor surely is that all these decisions themselves will create additional emotional problems for your relatives. Even in the first scenario they may be painfully aware of the fact that they *could* have given you a better life by continuing to take care of you, at whatever cost. Their sense of duty may not end when their duty ends; in any case they will not stop being concerned when the costs of acting on that concern are getting too high. In that case you could be tempted to think that you could help them to get rid of their feelings of guilt by getting rid of you. Still that seems an irrational response: these guilt feelings reflect a concern for your welfare, so presumably your relatives would not want to be relieved of those feelings by any act by which you sacrifice even more of that welfare. If they found out what motivated your treatment refusal or your suicide, their guilt feelings would only increase. It is hard to make general moral statements about such complicated patterns of mutually reflected feelings, but the default option surely should be that you firmly insist on your refusal, explaining that there is nothing they can do to change it.

That point also applies to the second scenario. If you decide that, having refused your relatives’ contributions to the fulfilment of your needs, you can only welcome death as your best remaining choice, you may also welcome the fact that you thereby will take an emotional load off their shoulders, at least in the long run. But your self-regarding reasons should in principle be sufficient for justifying that choice. Otherwise you are in danger of creating a new, perhaps even greater load.

Hardwig doesn’t accept this division of moral labour because he believes that such decisions ought to be collective ones, decisions for which all family members take equal responsibillity. That is a paradoxical position for him to take, because it overlooks the extent to which sharing this responsibility can be burdensome itself. The consequence could be that in order to relieve your relatives from the burden of care you saddle them with the even greater burden of being co-responsible for your death. It is not an ‘individualistic fantasy’ to suppose that in such conditions people should consult their relatives to the extent that this is possible without causing them undue additional distress, but should insist on taking full responsibility for their own decisions.

## Objections

I have argued that, under conditions that may actually occur, you can be required to forgo the offer of your intimates to give you the care you need, even though this leads to a situation in which your best prudential choice will be to refuse treatment or to end your own life. Although that second decision itself is self-regarding, it belongs to a package of decisions that is primarily other-regarding. Hence the package is still close enough to Hardwig’s ‘duty to die’ to invoke the same criticisms.

Let me consider those criticisms. A first objection is that we should be very reluctant to allow that people can be too much of a burden to each other, because people who are vulnerable to depressive disorders can catch on to the idea and start developing suicidal thoughts. Feeling oneself a burden to others is one of the most common and forceful motives for suicidal thoughts and acts,^18^ and often they reflect mistaken estimations of either the actual burdens or of the caregiver’s love and commitment, estimations that may be caused by a sense of one’s own unworthiness.^19^ On the other hand, however, these estimations may sometimes be correct or reasonable, hence they should not be considered to be a pathological symptom as such. To outlaw a public consideration of the possibility of a correct assessment altogether may not help people to calibrate their actual assessments, and therefore increase, rather than decrease the risk of their acting on mistaken ones. The bottom line, however, is that there is a truth of the matter, even though it may be advisable not to be too outspoken about it, at least not in all contexts.

A second objection is that actually there is no ‘truth of the matter’, or at least no authoritative or objective way to establish it. In considering whether the burdens of care are becoming too heavy, people will disagree about that, in particular the provider and the recipient of that care, and there will be no way to solve their disagreement.^20^ But the claim that ‘we have no way to solve our disagreement’ is ambiguous. It can mean that we have run out of arguments, because there is no determinate answer to the question in dispute. If this is the meaning of the claim, we need only to observe that there may be a zone of indeterminacy around the borderline we are trying to identify, but that doesn’t mean that there are no clear points at either side of the border outside that zone. If the answer to the question was fully indeterminate, there would, indeed, be not upper limit to the duty of care, but no lower limit either. A claim that the burdens are too heavy would then make no sense, but the claim that they are acceptable or proportional would make no sense either.

Because, as we saw, we should be wary of heroism, I am inclined to think that the grey area normally is not that large. Normally you should accept the (physical or financial) care you need *graciously*, and in particular if the care is offered by your loved ones, you should only consider refusing the offer if it is beyond reasonable doubt that you would be overtaxing them by accepting the offer. Or if this consideration only tips a balance mainly determined by other considerations. Much will depend on the details of the case.

The second possible meaning of the phrase ‘we have no way to solve our disagreement’ is that, if one of us is mistaken, there is nothing the other can do about it, because there is no procedure to arbitrate our conflict. If this is the objection, the answer is that no arbitration is needed, because we are not talking about enforceable duties. The people involved all have to make up their own minds about their own responsibilities. But they cannot determine their responsibilities. For if they have to make up their minds, rather than throw a dice, that presupposes that the decisions to be made on both sides can either be correct or mistaken, or at least reasonable or unreasonable, though there may be a grey area where they are not clearly either.

A third objection is that in setting up the problem as a conflict of interest between care-giver and care-taker, one discriminates against the elderly, because their lives are supposed to be of lesser value. Note, to begin with, that we are not talking about the value of their lives for others, but for themselves, a point often overlooked in such discussions. The main point to be made is then again that the problem of weighing the value of survival against the costs of care it requires others to pay, does not go away by denying its existence. By maintaining that the life of the elderly ‘has no price’, one necessarily leaves it to someone to pay the actual price. This is generally true of this rhetoric. Actually the problem arises for every case in which the burdens of care have to be distributed, not only when the care concerns elderly people. But a second point is that, other things being equal, both age and life-expectancy really *are* relevant. It is true that even very old people can still have genuine interests in survival. But not everyone has, and those who have can both consider that they have already had their ‘fair innings’,^21^ and that they only have a short time left to acquire more. Remember that we are not talking about the right to life which everybody has equally, let alone about social policies, but about responsible personal decision-making.

The most important objection is, finally, that within the family there should be no talk about duties of care and their upper limits, or about burdens that are ‘too heavy’, and that are attached to one family member’s valued existence. People’s interests are so interwoven with each other that even the burdens of care cannot be distributed, both the benefits and the costs will be internalized equally by all. However huge the burden of care, the burden of failing to provide it will be greater.^22^

Suppose that you are the person who needs to be cared for. The objection assumes that, if a moral requirement to refuse your relatives’ care at some point is recognized, it will be your care-giving partner, son or daughter who will complain that the burdens are becoming too heavy, and that your response to that claim should be that you unburden them, however grudgingly. Surely that is not a picture of a loving family. But if the burdens really are disproportionately high, you should be the first to recognize the fact. It could even be that your partner, son or daughter is quite willing to go to any length in order to give you the care you need. In that case surely you should at first be quite happy to be at the receiving end of that care and not be too scrupulous about ‘being a burden’. It is a normal condition of human beings, in particular at the beginning and the end of life, to be dependent on the care of others, and you should be particularly grateful if you have loving family members willing to take that task upon them.^23^ But it is quite another thing to be willing to let that task destroy their lives. Even if *they* don’t count the cost, you should. And even when they protest that they are fully prepared to go on paying that cost, they should understand that they cannot expect you to be equally willing to let them pay. Perhaps in a truly loving family people do not shrink from shouldering the burdens of care, however huge. Family may in that sense be a “haven in a heartless world”.^24^ But it is inconsistent to imagine these same people not to shrink from the same sacrifices to be made *for them*. The safe haven for the recipient might then become a heartless world for the provider.

Actually I do not agree that it is always praiseworthy to be prepared to give the care that is needed, irrespective of the costs. At some point it will be quite proper, in particular for caring children or friends, to remember that they have a life of their own to live. It may even be a duty they owe to themselves, if not to other family members, for example their own partner and children.

In most actual cases, you will only need a level of care that could overtax your intimates because of a physical and/or mental condition that will also corrode your capacities to contribute to the fulfilment of your own or other people’s basic interests and, partly for that very reason, cause you severe suffering.^25^ Hence in making up your mind about the discontinuation of your reliance on their endeavours when ending your life will be the corollary of that decision, you need not decide whether you would make the second decision without the first one, and even if you doubt whether you would, you need not explain this to them. In some cases an open conversation may be possible in which all parties are only sincerely trying to establish the facts as they are, eventually arriving at a decision that all can endorse. Though your relatives will start remonstrate with you by claiming that no amount of care will be ‘too much’ for them, they may allow themselves to be convinced by you when you say that there is much more at stake for them than for you, that they cannot expect you to stand aside and watch them sacrificing the ‘more’, and that this would even spoil the benefit they intended to create for you. But in many cases only by being less than fully transparent can we sufficiently respect each others’ feelings. That is not by itself a moral defect. From the fact that we cannot responsibly reveal some motive, it does not follow that we cannot responsibly be led by it. Even within fairly harmonious families there may be limits to the extent that the members can share their reasons.

I certainly do not wish to deny that your very thought of being a burden to your loved ones can itself be burdensome to them, even if your thought is fully true to the facts. One could even imagine a case in which you face the choice of devastating their life, either by the burdens of the care you need, or by their awareness that your refusal of that care has led you to end your life at the next step. Sometimes you could solve the dilemma by deciding to go to that nursing home after all, whatever your personal feelings, but if your relatives realize that this is really for you a fate worse than death, it is no solution. But the very fact that such an extreme case may present you with an unsolvable dilemma shows that the relevance for your decision of the fact of being a burden cannot simply be put aside by appealing to the nature of family life.

## Conclusion

I have argued that under any conditions that can be considered realistic at present, a person who is dependent on the care of her relatives cannot have a proper duty to end her life, either by refusing treatment or by suicide, a duty that she owes to her relatives. For they cannot at the same time have a duty to care for her and a claim-right to be relieved from the burdens of that care. It is possible, however, for her, in (hopefully) still rare circumstances to have decisive moral reasons to forgo that care, even if this decision brings her into a situation in which it will be preferable for her to die. This will not be a duty she owes to her relatives either, for in that case it would only exist as long as they did not waive their corresponding right, and she should not even bring her relatives into the position in which they would have to consider whether or not to waive that right. But it may be the right thing to do all the same.

This conclusion obviously has political implications. Shouldn’t the state do everything needed to prevent such circumstances from arising? Shouldn’t it at least do more than it does at present? *Could* it ever do enough? And if this is either beyond the capacities or the obligations of the state, shouldn’t we recognize obligations in this respect as citizens, more than we do at present? But it is beyond my present ambition to discuss these questions. They do not pre-empt the decisions that I have discussed in this paper. For we cannot say to a person who considers relieving her relatives from the burdens of care: wait until we have created a just society, then your problem will have disappeared.

## Footnotes

1 Landelijk Platform Ouderenmishandeling (2009). For a cross-national study, based on reports of GP’s, see Pivodic et al. (2014). These GP’s considered caregivers who provided long-term care at the end of life to be physically or emotionally overburdened in 28% (Belgium), 30% (the Netherlands), 35% (Spain) and 71% (Italy) of the cases. In 8% (Spain), 14% (Belgium), 36% (the Netherlands) and 43% (Italy) of the cases GP’s reported difficulties in covering care-related costs. Cf. Hardwig (2013, p. 115), for some details about the extent of family caregiving in the USA.

2 See the American case described by Hardwig (1997, p. 37).

3 Hardwig (1997, 2000, 2013). My discussion in this paper is restricted to the context of personal care. The possibility of a ‘duty to die’ has also been discussed in the context of the just distribution of scarce health care resources (cf. Battin 1987, 2000; Menzel 1990, 2000), interview with Mary Warnock in Beckford (2008).

4 Levvis and Levvis (2012).

5 Cf. Anderson (2000).

6 In some cultural environoments children are supposed to have fixed duties to their parents that are virtually independent of the behaviour of those parents. It is a difficulty question whether such social norms can have any ‘true’ moral authority. It is beyond the scope of this paper to discuss that question.

7 That a care-recipient may have to limit the demands made on a care-provider has occasionally been observed in discussions of the ethics of care (e.g. Tjong Tjin Tai 2006, p. 75; Kittay 2013, pp. 82–84) on being ‘overdemanding’.

8 The argument against Hardwig presented by Cholbi (2010), goes astray by failing to distinguish between these two senses of ‘duty’. He observes that according to Hardwig it can be ‘impermissible’ for you to fail to decide to die in your relatives’ interests, and objects that in that case they would have the right to kill you. But they clearly don’t have such a right. Therefore you have no ‘duty’ to die.

9 Let alone a duty corresponding to a right of them to kill him, as Cholbi (2010) suggests. Even if Oates would have had a strict duty to die, his comrades would, perhaps, not have wronged him by killing him, but that doesn’t mean that they would have had the right to kill him. For there might be reasons of public trust to prohibit such killings, or impersonal moral considerations. Or the mere fact that the law forbids them could have moral significance.

10 It is controversial whether implementing that decision by itself would have been a suicide. The actual way in which Oates implemented his decision, by walking into the blizzard in order to save his comrades the agony of the decision to leave him behind, certainly amounted to a suicide for altruistic reasons. But he didn’t have decisive moral reasons to act in that particular way, it was a supererogatory act.It has been objected that even in such cases one does not commit suicide because one would be all too happy when, miraculously, one survived. But that depends on a definition of suicide in terms of intention rather than foreknowledge and endorsement, that can be criticized (cf. Cholbi 2011, chap. 2), for an excellent discussion.

11 I therefore agree with Cholbi (2010) that there is an important difference between the case of Captain Oates and the cases to which Hardwig’s argument applies. But I don’t believe that the difference can be explained in terms of proportionality, see footnote 25 below.

12 Cf. English (1992). But I disagree with her view that children never ‘owe’ their parents anything.

13 Hardwig (2013), cf. Churchill (2000, p. 154).

14 Cf. Spellecy (2000, p. 210).

15 As proposed by Drebuschenko (2000).

16 Kittay (2013, p. 81).

17 It may be highly praiseworthy to do the right thing, if it requires making great sacrifices. That doesn’t mean, however, that we need a new category of ‘forced supererogation’, as proposed by Cohen (2013). Relieving your relatives from the burdens of care is one of his examples. Such acts may seem to be supererogatory because there is no strict duty, owed to someone else, to perform them.

18 Joiner (2005, p. 99).

19 If the perception of being a burden to others is prominent among the reasons for requesting euthanasia in the Netherlands in a particular case, physicians will, for such reasons, be reluctant to grant the request, Haverkate et al. (2000). Although the emotional and physicial burdens of care increase significantly towards the end of the care-recipient’s life, most care-providers do not perceive this as a problem because they consider caring a rewarding task, De Korte-Verhoef et al. (2014).

20 Callahan (2000), cf. Spellecy (2000). To some extent Hardwig invited this kind of criticism by insisting that the decision should be made by the family collectively.

21 Harris (1985, pp. 93–94).

22 Callahan (2000), Kass (1993, p. 271, cf. 281), footnote 1: Kass confesses to be tempted to save his family from the crushing burdens of care, but then asks: “What principle of family life am I enacting and endorsing with my ‘altruistic’ suicide?”

23 Kittay (2013, pp. 81–84) argues that “both refusing care and making incommensurate demands are strategies by which we deny our dependency on another.”

24 Callahan (2000).

25 If only for this reason Cholbi’s argument that, irrespective of the burdens, we always ask too much if we ask people to surrender the benefit of life, does not succeed, Cholbi (2011, pp. 109–111), cf. the appeal to a principle of proportionality to explain why you cannot have a duty to die in order to relieve your relatives from the burdens of care, Cholbi (2010), cf. also Drebuschenko (2000) for a similar but more limited claim. The benefits of life are not the same for everyone and for some are very modest. Cholbi’s view is a variant of what Hardwig calls ‘medical vitalism’, the idea that prolonging life is always the highest personal value. Interestingly, it is the essence of much of Daniel Callahan’s work to deny this, see for example Callahan (1987).
